# Supplementary material for: Geographical Variation of Honey Bee (Apis mellifera L. 1758) Populations in South-Eastern Morocco: A Geometric Morphometric Analysis
Source: Insects. 2022 Mar 15;13(3):288. doi: 10.3390/insects13030288 (PMC8953116; doi:10.3390/insects13030288)
Supplement: Supplementary file 1 [file insects-13-00288-s001.zip › insects-1602074-supplementary.pdf]

Supplementary Table S1. Locations where honey bee samples were obtained from southeastern Morocco.

Code	Area	Location	Hive type	Latitude	Longitude
1	Errachidia	Aoufous	modern	31.689083	-4.17165
2	Errachidia	Ibarbatene	modern	31.979092	-4.476538
4	Errachidia	Ksar Ouled Saaidane	modern	31.300800	-4.324667
5	Errachidia	Ksar Ouled Saaidane	modern	31.300800	-4.324667
6	Errachidia	Merzouga 1	modern	31.071838	-4.035732
7	Errachidia	Merzouga 1	modern	31.071838	-4.035732
8	Errachidia	Merzouga 1	modern	31.071838	-4.035732
9	Errachidia	Merzouga 1	modern	31.071838	-4.035732
10	Errachidia	Merzouga 1	modern	31.071838	-4.035732
11	Errachidia	Merzouga 1	modern	31.071838	-4.035732
12	Errachidia	Merzouga 2	modern	31.072400	-4.035383
13	Errachidia	Merzouga 2	modern	31.072400	-4.035383
14	Errachidia	Merzouga 2	modern	31.072400	-4.035383
15	Errachidia	Merzouga 2	modern	31.072400	-4.035383
16	Errachidia	Merzouga 2	modern	31.072400	-4.035383
17	Errachidia	Rissani	modern	31.278083	-4.203867
18	Errachidia	Rissani	modern	31.278083	-4.203867
19	Errachidia	Tahesnounte	modern	31.345100	-4.278433
20	Errachidia	Tahesnounte	modern	31.345100	-4.278433
21	Errachidia	Tighejdet	modern	32.234156	-4.63233
24	Ouarzazate	Ait Zineb	modern	30.982400	-7.148517
25	Ouarzazate	Ait Zineb	modern	30.982400	-7.148517
26	Ouarzazate	Ait Zineb	modern	30.982400	-7.148517
27	Ouarzazate	Amekchoud 1	traditional	31.219417	-6.585267
28	Ouarzazate	Amekchoud 1	traditional	31.219417	-6.585267
29	Ouarzazate	Amekchoud 1	traditional	31.219417	-6.585267
30	Ouarzazate	Amekchoud 2	modern	31.221217	-6.581833
31	Ouarzazate	Amekchoud 2	modern	31.221217	-6.581833
32	Ouarzazate	Amekchoud 2	traditional	31.221217	-6.581833
33	Ouarzazate	Asserssa	modern	30.526633	-7.15195
34	Ouarzazate	Asserssa	modern	30.526633	-7.15195
35	Ouarzazate	Asserssa	modern	30.526633	-7.15195
36	Ouarzazate	Halouqte	modern	30.705867	-7.307917
37	Ouarzazate	Halouqte	modern	30.705867	-7.307917
38	Ouarzazate	Halouqte	modern	30.705867	-7.307917
39	Ouarzazate	Iminoulaoune	modern	31.299800	-6.500117
40	Ouarzazate	Iminoulaoune	modern	31.299800	-6.500117
41	Ouarzazate	Iminoulaoune	modern	31.299800	-6.500117
42	Ouarzazate	Ouled Arbia	traditional	31.047533	-6.594533
43	Ouarzazate	Ouled Ibrahim	modern	31.111050	-6.581467
44	Ouarzazate	Ouled Ibrahim	modern	31.111050	-6.581467
45	Ouarzazate	Ouled Ibrahim	modern	31.111050	-6.581467
46	Ouarzazate	Ouarzazate	modern	30.918683	-6.91995
47	Ouarzazate	Ouarzazate	modern	30.918683	-6.91995
48	Ouarzazate	Ouarzazate	modern	30.918683	-6.91995
49	Ouarzazate	Timsal	modern	31.271333	-7.13825
50	Ouarzazate	Timsal	traditional	31.271333	-7.13825
51	Ouarzazate	Timsal	modern	31.271333	-7.13825
52	Ouarzazate	Timsal	traditional	31.271333	-7.13825

Supplementary Table S1. (Continued)

Code	Province	Region	Hive type	Latitude	Longitude
53	Ouarzazate	Timsal	modern	31.271333	-7.13825
54	Ouarzazate	Timsal	traditional	31.271333	-7.13825
3	Tinghir	Ksar Lbour Tinejdad	modern	31.446600	-5.169717
22	Tinghir	Tinejdad	modern	31.512387	-5.027543
23	Tinghir	Tinejdad	modern	31.512387	-5.027543
55	Tinghir	Ait Ibriren	modern	31.450800	-6.012117
56	Tinghir	Ait Ibriren	modern	31.450800	-6.012117
57	Tinghir	Ait Ali Ou Brahim	modern	31.469850	-5.39865
58	Tinghir	Ait Ali Ou Brahim	modern	31.469850	-5.39865
59	Tinghir	Anou n'Ou'achcha	modern	31.292483	-5.651533
60	Tinghir	Anou n'Ou'achcha	modern	31.292483	-5.651533
61	Tinghir	Anou n'Ou'achcha	modern	31.292483	-5.651533
62	Tinghir	Aït Toumort 1	traditional	31.400972	-6.274639
63	Tinghir	Aït Toumort 1	modern	31.400972	-6.274639
64	Tinghir	Aït Toumort 1	modern	31.400972	-6.274639
65	Tinghir	Aït Toumort 2	traditional	31.451283	-6.326083
66	Tinghir	Aït Toumort 2	traditional	31.451283	-6.326083
67	Tinghir	Aït Toumort 2	traditional	31.451283	-6.326083
68	Tinghir	Ikisse Amezdar	modern	31.314600	-5.640567
69	Tinghir	Ikisse Amezdar	modern	31.314600	-5.640567
70	Tinghir	Ikisse Amezdar	modern	31.314600	-5.640567
71	Tinghir	Ikisse Amezdar	modern	31.314600	-5.640567
72	Tinghir	Souk El-Khémis Dades	traditional	31.306167	-6.033783
73	Tinghir	Souk El-Khémis Dades	traditional	31.306167	-6.033783
74	Tinghir	Souk El-Khémis Dades	traditional	31.306167	-6.033783
75	Tinghir	Tilmi	modern	31.794867	-5.776000
76	Tinghir	Tilmi	modern	31.794867	-5.776000
77	Tinghir	Tilmi	modern	31.794867	-5.776000
78	Tinghir	Timit	traditional	31.290150	-5.554917
79	Tinghir	Timit	traditional	31.290150	-5.554917
80	Tinghir	Timit	traditional	31.290150	-5.554917
81	Tinghir	Timit	traditional	31.290150	-5.554917
82	Tinghir	Tourza	modern	31.234867	-5.261467
83	Tinghir	Tourza	modern	31.234867	-5.261467
84	Tinghir	Tourza	modern	31.234867	-5.261467
85	Zagora	El Gloa	modern	30.274917	-6.476583
86	Zagora	El Gloa	modern	30.274917	-6.476583
87	Zagora	El Merja	modern	30.183200	-6.40225
88	Zagora	El Merja	modern	30.183200	-6.40225
89	Zagora	Faïja	modern	30.176033	-6.123933
90	Zagora	Ighereghar	modern	30.678450	-6.293967
91	Zagora	Ighereghar	modern	30.678450	-6.293967
92	Zagora	Ighereghar	modern	30.678450	-6.293967
93	Zagora	Ighereghar	modern	30.678450	-6.293967
94	Zagora	Ighereghar	modern	30.678450	-6.293967
95	Zagora	Intliten	modern	30.746233	-6.436647
96	Zagora	Intliten	modern	30.746233	-6.436647
97	Zagora	Intliten	modern	30.746233	-6.436647

Supplementary Table S1. (Continued)

Code	Province	Region	Hive type	Latitude	Longitude
98	Zagora	Intliten	modern	30.746233	-6.436647
99	Zagora	Intliten	modern	30.746233	-6.436647
100	Zagora	Tamnougalt 1	modern	30.675000	-6.396767
101	Zagora	Tamnougalt 1	modern	30.675000	-6.396767
102	Zagora	Tamnougalt 1	modern	30.675000	-6.396767
103	Zagora	Tamnougalt 2	modern	30.678190	-6.386156
104	Zagora	Tamnougalt 2	modern	30.678190	-6.386156
105	Zagora	Tamnougalt 2	modern	30.678190	-6.386156
106	Zagora	Tighremet	modern	30.854100	-5.838850
107	Zagora	Tighremet	modern	30.854100	-5.838850
108	Zagora	Tighremet	modern	30.854100	-5.838850
109	Zagora	Tighremet	modern	30.854100	-5.838850
110	Zagora	Tighremet	modern	30.854100	-5.838850
